# A narrative inquiry into healthcare staff resilience and the sustainability of Quality Improvement implementation efforts during Covid-19

**DOI:** 10.1186/s12913-023-09190-4

**Published:** 2023-02-24

**Authors:** Shannon Hughes Spence, Zuneera Khurshid, Maureen Flynn, John Fitzsimons, Aoife De Brún

**Affiliations:** 1 Education and Innovation in Health Systems (UCD IRIS Centre), University College Dublin (UCD) Centre for Interdisciplinary Research UCD School of Nursing, Midwifery and Health Systems University College Dublin, Dublin, Ireland; 2 Improvement Academy, Bradford Institute for Health Research, Bradford Teaching Hospitals NHS Foundation Trust, Duckworth Lane, Bradford, UK; 3 Office of the Nursing and Midwifery Services Director, National Quality and Patient Safety Directorate, Health Service Executive (HSE), Dublin, Ireland; 4 Temple Street and Clinical Director for Quality Improvement, Health Service Executive (HSE), Children’s Health Ireland, National Quality and Patient Safety Directorate, Dublin, Ireland

**Keywords:** Quality Improvement, Sustainability, Resilience, Covid-19, Healthcare staff experience

## Abstract

**Background:**

Recent research, which explored the use of Quality Improvement (QI) methods in the Covid-19 pandemic response, found that Quality Improvement principles were utilised during the crisis management period, albeit without direct intention. Following on from this work, the aim of this paper extends that study by investigating the sustainability and resilience of not only the changes implemented by healthcare staff during Covid-19 in Ireland, but the resilience of the wellbeing of healthcare staff themselves through the various waves of Covid-19.

**Methods:**

To explore healthcare staffs experience of Quality Improvement and the sustainability and resilience of both Quality Improvement initiatives and healthcare staff, a qualitative design was implemented. Semi-structured interviews took place online over Zoom with 11 healthcare staff members from the Irish healthcare service in the Spring of 2022. An analysis of the narratives was conducted using thematic analysis supported by NVivo12.

**Results:**

Four key themes were evident from the data: (i) From fear to exhaustion; (ii) maintaining person-centred approaches to care; (iii) Covid-19 as a medium for change, and; (iv) staff resilience and appetite for Quality Improvement.

**Discussion:**

The results of this work identified three key learnings; (i) integrating learning into policies and practice: (ii) the role of collective leadership and devolving/sharing power; and (iii) key drivers/factors that promote sustainability of QI interventions. Despite the challenges in recruitment of research participants experienced during the pandemic, a narrative approach supported the collation of rich and nuanced insights into the experiences of healthcare staff during this time.

**Conclusion:**

A growing body of literature currently exists on how healthcare staff felt during the Covid-19 pandemic. However, as the waves of Covid-19 have declined, it is vital to examine how the feelings of burnout and disillusionment will affect engagement with Quality Improvement in the future. It is also worth noting and examining the feeling of purpose and pride participants expressed from working through the Covid-19 pandemic. This study has helped to address this gap.

**Supplementary Information:**

The online version contains supplementary material available at 10.1186/s12913-023-09190-4.

## Introduction

Although a sense of duty, pride and purpose has been felt amongst healthcare staff who have worked through the Covid-19 pandemic [1], there has been an increase of stress, burnout, depression, substance misuse and suicide across all groups of healthcare workers in several countries over the last number of years [1]. The mental health and psychological wellbeing of healthcare workers was a major healthcare issue even before the Covid-19 pandemic [2, 3],with burnout occurring at 35% to 54% among nurses and physicians [4]. Resilience, therefore, has come to the fore of discussions surrounding healthcare workers. Resilience has often been defined as the ability to positively adapt to challenging and traumatic experiences [5]. There is a link between lower resilience, burnout and poorer quality of care and patient outcomes [3]. As shown in an integrated review examining healthcare workers resilience levels during the Covid-19 pandemic, healthcare workers around the world are reporting low-to-moderate resilience levels. It is therefore essential that health leaders, healthcare organisations and governments prioritise support and resources for healthcare workers. The pandemic has only exacerbated feelings of anxiety, stress, depression and burnout [6–8]. With inadequate staffing levels, inexperienced staff, and an increasing number of critically ill patients, many nursing staff during the Covid-19 pandemic and prior pandemics, reported feeling broken, crying on the way to work, in work and after their shift had finished, as highlighted in a recent systematic review and qualitative meta-synthesis [2]. Staff experienced a range of negative impacts such as sleep disturbance, panic attacks, feelings of guilt, grief, and dread [2].

Research has revealed differences in individual-level resilience. In a study of resilience among UK and Irish healthcare workers, both samples of frontline workers had similar levels of resilience and burnout, with UK-based workers having significantly lower wellbeing. This discrepancy was attributed to the differing government responses between Ireland and the UK to the Covid-19 pandemic [9]. While the Irish government acted almost immediately in bringing about restrictions in an attempt to curb the spread of Covid-19, the government response from the UK was not as timely. Further, the rapid rise of Covid-19 cases in the UK was not replicated as quickly in Ireland. On the 11^th^of March 2020 the Irish government issued advice to close schools, colleges, and universities, and limit public gatherings to under 100 people in the case of indoor events, and 500 for outside events. The UK however did not implement such regulations until late March. Both the late response on behalf of the UK government and the increasing amount of Covid-19 cases may have contributed to UK-based workers reporting higher levels of burnout, and lower levels of resilience and wellbeing, compared with that of their Irish counterparts [9]. The response to Covid-19 has been described as being more akin to a marathon than a sprint and working at or beyond full capacity is unsustainable in the medium and long term [10].

With frontline workers and the frontline health services having come under an unprecedented level of pressure during Covid-19, a focus on improvement is essential to orientate the planning and delivery of healthcare away from crisis management to proactive ongoing service improvement [11]. Increasingly QI is considered a crucial part of healthcare workers’ role. Within an Irish context, the Irish health service, the Health Service Executive (HSE) promotes and encourages healthcare workers to engage with QI through the National Quality Improvement Team [12]. The National Quality Improvement Team developed the Quality Improvement Toolkit as a tangible to facilitate staff training in QI. The toolkit contains several tools that can be applied to a number of QI projects, aiding healthcare workers in their experience and training with QI [13].

There are multiple QI definitions and methods used globally, and within Ireland, with the Institute for Health Improvement and Lean amongst the most common. This study has therefore adopted the Institute for Health Improvement’s definition of Quality Improvement (QI) due to its focus on the Plan-Do-Study-Act (PDSA) cycle. Thus, QI is defined as “rapid-cycle testing in order to learn which interventions, in which contexts, can predictably produce improvements” ([14] p.5). Further, QI offers systematic approaches that can aid in adapting to change and is thought to be a useful asset in responding to a crisis such as the Covid-19 pandemic [15]. As a result of successful implementation of change generated through QI during Covid-19, an opportunity exists to build on the widespread change that has been led by healthcare staff to embed the rigour of QI [16, 17]. For example, when QI initiatives were put forward, they may have taken a significant amount of time to be approval. However, during the Covid-19 pandemic, change was implemented at a much more accelerated rate. Recent research, which explored the use of QI methods in the Covid-19 pandemic response, found that QI principles were utilised during the crisis management period, albeit unintentionally [11]. For example, although QI practices and principles were evident throughout healthcare workers stories and actions (such as making small changes, testing changes, learning, reflecting and improving), it was only upon reflection that staff members identified that they were engaging in QI practices [17]. Further, it was highlighted that Covid-19 “eliminated some traditional barriers to change” ([17], p.1) such as working within strict siloes and bureaucratic heirarchies [17], “thus highlighting a need to sustain these positive changes into routine practice to develop an adaptive healthcare system receptive to QI” ([17], p.1).

Sustainability within healthcare can be defined as “the continued use of program components and activities for the continued achievement of desirable program and population outcomes” ([18] p.2060). With finite economic resources, the most pressing threat to health service sustainability has often been identified as financial. However, healthcare delivery also takes place within important social and environmental contexts [19]. With healthcare professionals under increased pressure to go beyond just delivering exceptional patient care within finite resources and continuing to manage their exhaustion from working through the Covid-19 pandemic, it is vital that QI is understood as a fundamental aspect of healthcare provision, and not an additional add-on for healthcare staff. By understanding that QI is a foundational aspect of healthcare delivery within the Irish healthcare system, this understanding ensures that sustainability is a key component of QI, which is essential in maintaining the resilience of both the health systems and healthcare workers [18]. Further, due to the pressure on healthcare workers to deliver changes, and the lack of research into if/how well these changes have been sustained given the increased exhaustion and burnout among staff, this research into the sustainability and resilience of not only healthcare workers, but QI projects, is timely.

Following on from previous research exploring staff experiences of QI during the Covid-19 pandemic, the aim of this paper was to explore the sustainability and resilience of not only the QI changes implemented by healthcare staff during Covid-19 in Ireland, but the resilience of the healthcare staff themselves throughout the various waves of the Covid-19 pandemic.

## Methods

### Covid-19: The Irish context

Throughout several significant waves of Covid-19 in Ireland from 2020 to 2022, various degrees of restrictions were implemented and lifted throughout the two years. Although many aspects of life seemed to slow down, the health service came under increasing pressure as it attempted to cope with the increase of patients being admitted with severe cases of Covid-19 [20]. Thus, the longitudinal nature of this research seeks to explore the resilience of healthcare staff who worked throughout the pandemic and their capacity to engage with, and sustain, QI initiatives, as data was initially collected at the beginning of the Covid-19 pandemic in 2020 [11] and then again in 2022.

### Qualitative narrative approach

Narrative inquiry was employed to discover the real life experiences of healthcare workers through their own words. As with other forms of qualitative research, narrative inquiry allows thematic patterns to emerge when exploring the lived experiences of individuals [21]. Participants were asked to share their experience of working and implementing one or more specific changes during Covid-19. Participants began by explaining their service before Covid-19, during, and ‘after’ as the waves lessened, following a somewhat sequential timeline. Narrative inquiry is not simply storytelling, it is a method of inquiry that utilises storytelling to discover nuance as it provides the opportunity for dialogue and cyclical reflection [22]. Healthcare and nursing practices are dynamic processes underpinned by continuous interaction of human thought and behaviour that feed into personal, social and material environments [18]. This allows narrative inquiry as a methodology in healthcare research to be exceptionally useful to uncover the detail of healthcare workers experiences, diving into the complexity of care, the “messy” events that can occur in the workplace, and service weaknesses [23]. Through the use of semi-structured, probes where used when necessary to elicit information regarding concepts of resilience and sustainability to address the context of the narrative. Such probing questions consisted of “was there anything different about how you worked with colleagues to implement the change/initiative as compared to how you usually work together?”, and “has this process/initiative continued post-Covid? Has it changed in anyway? If so, how? Why do you say this?”.

Several studies investigating the impact of Covid-19 on healthcare workers have highlighted the need for qualitative inquiry into the experiences of healthcare workers during the pandemic. Implementing a narrative analysis allows for healthcare workers to discuss their experience which may encourage them to appreciate the perspectives of others, potentially leading to a collective question of “what should we now go on to do?” ([20], p.3).

The process of a narrative inquiry included the analysis of the text (in this case, the transcribed semi-structured interviews) within the historical, social and cultural context that they occurred from the participants perspectives [24, 25].

### Sampling strategy

**Fig. 1:**
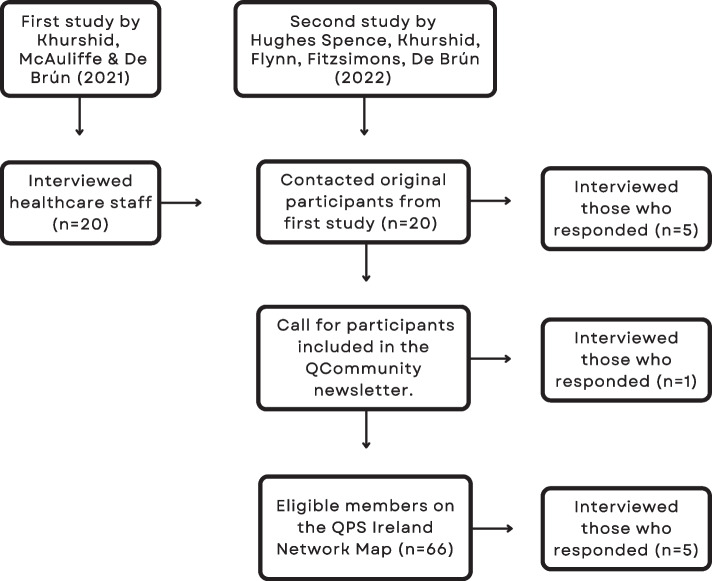
Recruitment strategy flowchart

Staff working within a healthcare setting during Covid-19 who had experience of working with QI were recruited for this study. Participants were recruited via three different recruitment avenues: (i) re-interviewing previous participants; (ii) a recruitment call through the *Q*Community of Ireland tea time catch up infographic summary update, and the Quality and Patient Safety (QPS) Ireland Network Map [26] and; (iii) a recruitment call via social media (Twitter). A recruitment strategy flowchart can be seen in Fig. 1.

The eligibility criteria for recruitment consisted of people who worked in healthcare during the Covid-19 pandemic and who had experience of QI. Recruitment phase one consisted of re-interviewing research participants from the first round of data collection (*n*= 20). The first round of data collection refers to a previous study carried out by some of the authors [17]. Thus, the first recruitment phase was to retarget the individuals who were interviewed for the previous study. Those previous participants were contacted in March 2022 requesting their participation in a follow up interview. Those who responded were re-interviewed (*n* = 5). The second phase of recruitment consisted of a call for participants was included in the *Q*Community team time infographic update that was sent out to its members on April 15th. One participant was recruited through the *Q*Community Newsletter. The *Q*Community is a community of thousands of people within the UK and Ireland that work to improve the quality of health and care. It is delivered by the Health Foundation [27]. The QPS Ireland Network Map is an online tool that maps and connects those who work within healthcare in Ireland who have an interest in QI [28]. Using the QPS Ireland Network Map, out of the 203 members on map, 71 people had their email addresses accessible on their profile to other map members. Out of the 71 who had their email addresses available, 66 were eligible to participate. An email invitation was sent followed by a follow-up/reminder two weeks later and five people were interviewed as a result. The last phase of recruitment consisted of posting in three special interest groups in the community forum of the *Q*Community Special Interest Group, a recruitment flyer being shared on social media (Twitter) and a recruitment call on Hexitime, an online platform for those working in healthcare to exchange ideas and time [29]. No participants were recruited using this method.

### Data collection methods

Narrative style semi-structured interviews were conducted with each of the eleven participants. The interview guide consisted of questions covering topics such as if/how participants ways of working changed during the pandemic, if the participant considered the changes and initiatives that were implemented successful, how staff are currently feeling, and what do staff need at the moment to effectively manage and sustain quality and improvement initiatives. Written and verbal consent was obtained from each participant prior to the interview beginning. The interviews were conducted remotely over Zoom throughout March, April and May 2022. The interviews were audio recorded, transcribed verbatim and pseudonymised to ensure the participants identities would remain anonymous.

### Data analysis

The qualitative coding software NVivo was used to support data analysis [30]. A qualitative thematic analysis was employed to analyse the interview data through an analysis of narrative. The distinction between narrative analysis and an analysis of narrative has distinguished by the following definitions; a narrative analysis refers to the gathering of descriptions of actions and events as data which the researcher uses to “generate stories though a process of emplotment” ([31] p.220) whereas the analysis of narrative views stories as data which are then analysed by the researcher for reoccurring themes, which was employed within this research. The stages of the thematic analysis are presented in Table 1 and have been adopted from previous thematic analysis of narrative inquiry [32].

**Table 1:** Stages of thematic analysis

Stage one	A preliminary reading and coding of the narrative was conducted to gain familiarity with the content. Initial descriptive findings were presented to two other authors
Stage two	Connections within the data were found by reading the narrative several times and reflecting upon the content
Stage three	With the aid of visual mind mapping techniques, overarching categories were developed
Stage four	The narratives were revisited again and coded to the emerging themes

## Results

In total, 11 participants took part in an interview with the lead researcher. Interviews lasted between 23 to 45 min, with an average of 30 min. Table 2 displays the sample characteristics of the participants.

**Table 2:** Participants sample characteristics

**Characteristic**	N
Number of participants	11
Pandemic redeployment experience	5
Leadership/supervision/management responsibilities	6
Previous exposure to service improvements	11
Have since left direct healthcare practice	2
In a patient-facing role	5
**Years of experience**	
15 – 20 years	2
21 – 26 years	5
27 – 32 years	2
33 – 38 years	2
**Area of expertise**	
Tom: Cardiac physiologist	1
Sarah: Community healthcare manager	1
Jake: Dentist	1
David: Consultant	1
Stacey and Daniel: Physiotherapists	2
Sabrina: Covid contact tracing staff member	1
Michelle: Quality and patient safety educator	1
Maggie: Quality improvement facilitator	1
Gavin: Clinical audit facilitator	1
Clara: Palliative care nurse	1

Consistent with the narrative inquiry approach, each of the interviews were distilled in a narrative summary which detailed each of the participants’ experiences.

Four key themes were evident from the data: (i) From fear to exhaustion; (ii) maintaining person-centred approaches to care; (iii) Covid-19 as a medium for change; and (iv) staff pride, resilience, and appetite for QI.

### From fear to exhaustion

The experiences articulated within the participants stories’ suggest that when the initial adrenaline and fear lessened as the pandemic went on, it was replaced by feelings of exhaustion, not just from working longer hours to manage the crisis, but also from the cumulative impact of the on-going and systematic issues that pre-dated the pandemic such as limited resources, waiting lists and being short staffed.

Clara shared how feelings of fear, anxiety, stress, and uncertainty were felt by healthcare staff at the beginning of the Covid-19 pandemic. Clara further explained how inadequate staffing levels exacerbated feelings of burnout amongst workers.

*“There was days when there was no staff available. And there was one girl I was working with and she'd been on for nine days and she was fit to fall on her feet with exhaustion and she herself was feeling unwell, but there's nobody (else) there to do it. That was really stressful”.*

Tom emphasised how healthcare workers are exhausted, although not necessarily just from working throughout the Covid-19 pandemic.

“I don't think they're necessarily exhausted from Covid. I think they're exhausted because of our situation of being short staffed”.

Gavin mentioned the “*legacy of exhaustion*” amongst healthcare staff that accumulated during the Covid-19 pandemic response.

“They're exhausted. They're stressed out. You know the way everybody says ‘oh they're exhausted’, but in actual fact you can see it”.

Stacey shared how frontline healthcare workers are experiencing feelings of exhaustion and are under pressure to return to actively engaging in QI.

*“They’re all still tired, they're all still really exhausted. I think now people are beginning to realise just how tired they are, and yet we have pressures to kind of get back to improving health care. And everyone's a little bit like ‘Oh God I'm not sure I have the headspace for this, I'm exhausted’”.*

Sabrina described the feelings amongst healthcare staff as “*a bit of a roller coaster*”. Although the initial fear that existed at the beginning of the Covid-19 pandemic has lessened, the intensity and pressure felt by frontline workers has not decreased.

*“For some frontline services and certainly within the hospitals, there's a sense that they’ve never really got that break, you know, they haven't really had that recovery time because the intensity is still there in a different way and now they’re back to the old issues of trolleys and waiting lists. There's a lot of burnout and there's a lot of disillusionment among staff, but not everywhere”.*

Jake highlighted how he is “*sort of struggling just to kind of keep breathing at the moment, so anything new … is a bit disruptive*”. With this in mind, consideration for the current capacity of healthcare workers to engage with new initiatives must be taken into account, as highlighted by some participants.

### Maintaining person-centred approaches to care

There was a fundamental and collective need to maintain care for patients during Covid-19 that would minimise the risk of spreading the virus, as shown by the participant quotes below and the implementation of technology-based solutions. As a result, many organisations implemented QI initiatives, including a TeleHealth platform, to deliver person-centred care.

Despite the risk of Covid-19 and the changes that were made to care delivery processes as a result, there was a continued focus on the importance of person-centred care expressed by the participants. Clara shared how she did not want to leave or abandon her patients and ensured someone remained at the bedside when patients were dying, sometimes resulting in nurses who questioned if they had done enough for their patient. The urgency and quick implementation of QI initiatives were vital in continuing person-centred care, such as the quick move to TeleHealth platforms. Daniel shared how his department had to quickly move to a TeleHealth format for their respiratory outpatients.

*“So what we had to do when Covid came initially, we had to adapt our TeleHealth service, so our outpatients service changed. This all seemed to happen overnight, but I suppose there was a lot of depth and planning to it albeit it fast”.*

Similarly Stacey highlighted that there was a focus on not wanting to disrupt services for patients. Stacey shared how departments within her organisation educated their patients how to use TeleHealth platforms.

*“Even people who were maybe not tech savvy or didn't think they were tech savvy, we put in lots of systems to support them, so we might bring them in for one or two sessions. And the one or two sessions might be focused on building up their technical capabilities, as well as maybe looking at them from a physio point of view, so, then they can utilize all of the kind of technically assisted services that are there”.*

However, duplicitous speak around the value of person centred care in organisations was highlighted by Gavin, underscoring the importance of authenticity in leadership.

**“***The hospitals all over the country have ‘we're patient centered’ as a kind of value that they cherish dearly. It actually got senior people to admit, behind closed doors, at times, ‘really we’re an awful lot better without visitors, aren’t we?’. And you kind of go ‘you can't say that’. But that to me clarified a duplicitous speak in organisations. And so I think frontline staff began to realise that what is said might not be the value in action”.*

### Covid as a medium for change

Although Covid-19 was undoubtedly a huge challenge for healthcare workers, it provided an opportunity to rapidly develop change initiatives, trial them and make adjustments where necessary. This willingness to change was attributed to the united common goal of combatting the virus, allowing hierarchies to flatten and bureaucratic red tape to by bypassed. However, within some organisations, such barriers to change are beginning to re-establish themselves, despite the desire to maintain a more flattened hierarchy, as articulated by the participants.

Healthcare staff quickly recognised the power of Covid-19 as a means to implement QI and positive changes. David revealed how he and others within his field have been trying to encourage routine mask wearing for years when there is a risk of a respiratory virus transmission.

*“We’ve been trying to get people to do that (wear masks) for years. When there's a risk of respiratory virus transmission or when somebody has respiratory symptoms.. It's been always been very, very difficult to translate that into large scale behavior change. Because of the necessity of the pandemic, it has now become the norm”.*

Maggie indicated that a digital nurse tracking system that had previously been “*in the works pre-Covid*” was fast tracked due to Covid-19 as “*it was an agile move to support an evolving situation*”. This system allowed for management to see which staff member was absent.

Sarah conveyed how a referral pathway project which connects General Practitioners (GP) and primary care services was started in 2019. However, she described the experience of her team as.

*“[coming up] against a little bit of a brick wall, where we were told from our communications department that we couldn't actually connect the two. But at the early days of Covid, the referral pathway was established and every GP in the country had access through their (referral pathway project name) service”.*

The participants attributed the successful and sustained initiatives to the QI methodology, such as the Institute for Healthcare Improvement model, used when planning the project and the QI culture embedded into their organisations.

Daniel shared that he believed that “*having a culture of QI and a culture of ‘let's try and test it change and see how we go’ was very much embraced”.* Similarly, Maggie expressed how using early adopter theory and QI methodology allowed for their initiative to *“spread bigger”.*

Stacey also attributed being a “*change ready*” organisation to the success of the QI initiative.

*“We try and always be a change ready environment, that's the culture we try and embed. So we try and look around corners and see down the road for what's potentially coming. So I think because we have that kind of change ready culture, we were a culture who had improvement methodology embedded in how we work”.*

There has been significant QI changes within the participants’ organisations during the pandemic. This is partly attributed to the decrease in barriers to change. However, there is a fear that the bureaucratic red tape and hierarchies, or the barriers to change, have slowly crept back to what they were pre-Covid for a variety of reasons, slowing the pace of change.

Daniel mentioned how change could take a significant amount of time pre-Covid, whereas with Covid, change happened rapidly. Now, Daniel fears that the willingness to trial changes has been decreasing.

*“I just hope, like before you could have been a year waiting to get something approved. Then, with Covid it went that you were able to get it approved literally within the week, whereas now, we have gone back till about six or seven months, so I just hope that they’re (senior management) as open to trialling things”.*

Likewise, Tom expressed how Covid allowed for the ability to rapidly develop initiatives and how organisations have begun to revert back into hierarchical structures that existed in pre-Covid times.

*“I think one of the things we are starting to lose that we had during Covid is the ability to rapidly develop things. We’ve gone back to the hierarchy thing a little bit. It's hard to articulate that but I think people start to bring their agendas back in again, you know, ‘that's not a priority for me, so therefore we’ll push it down the road for six months’. I think that becomes a bit of a challenge as well, and once that happens, then you get the hierarchy back again... And I think we still work in silos, we still work in professional silos and people are trying to advance their profession and maybe holding back change.”*

On a similar note, David highlights how hierarchies and inefficient management approaches that existed before Covid, although momentarily set aside to allow for necessary rapid change, have started to return.

*“The problems in terms of hierarchies and outdated management approaches that were there before the pandemic… to a certain extent, kind of got pushed to one side. It was kind of a case of ‘we've got more important things to think about right now’. You have that sense of hope but it's balanced out with this sense of ‘oh god, all that stuff that we had to deal with before, it's still there’.”*

Further, Gavin explicitly mentioned how healthcare workers need hierarchical management to grant permission to continue to engage in QI initiatives.

*“They need people to come along and tell them that they're doing a good job, and not just kind of lip service but actual tangible resources and making their job easier and they need acknowledgement and they need the space to do these things, so they need the hierarchy to give them permission to do these things.”*

Similarly Sabrina recognised how quickly change was implemented during Covid-19. However, within Sabrina’s organisation, the willingness to rapidly develop and trial initiatives has remained.

*“There were all these processes and hoops you had to jump through before anything could change. Whereas during Covid, in the early days everything had to change so quickly and people just really bought into that I think at that time, which was, I think, a huge positive for service improvement, and I think that has stayed. It’s settled a bit, but I think that has stayed to an extent, certainly in terms of new ways of working”.*

Now that staff have seen how quickly initiatives can be implemented, there is a level of motivation to continue to push for change that happens at a faster pace. Michelle expressed such a sentiment.

***“****We maybe bypassed a lot of bureaucracy in that time. We escalated a lot of things and doors were open to us to enable us to do the job. I think we question things more now and say ‘well look, why does it take six months to get something approved when it could be done shorter?’”.*

### Staff pride, resilience and appetite for QI

The pride healthcare staff took from working throughout the Covid-19 pandemic was evident amongst the participant, alongside their resilience. Although participants shared that there is an appetite for QI, they were quick to highlight how the Covid-19 pandemic has impacted healthcare staffs’ morale and ability to engage in what is seen as extra work.

Participants expressed a great sense of pride for the role they played during the Covid-19 pandemic. Clara believed that.

*“the people that stood up when it mattered, I feel that they will have learned a lot from experience. It's amazing how adaptable we can be. It's amazing how streamlined we can be when we think about what is the priority for patients and residents”.*

Similarly, Maggie expressed how her and her teammates felt privileged to be able to work together and would often think that the experience would be something that they would tell their future grandchildren about.

Gavin mentioned how people within his organisation who championed QI used Covid-19 as a chance to showcase the relevance of QI, particularly its applicability in a crisis situation.

*“We would have improvement champions across the organisation informally noted, as opposed to formal positions. And those people used the external environment constraints to maximise their improvement effort”.*

Daniel hopes that policymakers will realise the change that was achieved during Covid-19 and the strides that were taken around QI.

*“I just hope that the policymakers will actually see it going forward and about the importance because such good change has been done with Covid. So I just hope we don't go back to that hierarchy where all ideas are kind of shut down, and I do just hope that they continue to support the QI going forward, because it is the only way that people will improve standards”.*

Gavin highlighted that although the Covid-19 waves have lessened, a new sense of dread and stress is emerging as staff begin to face pre-existing issues that were side-lined during the pandemic. Pre-existing issues within the health service are thus coming back into focus (availability of resources, difficulty with recruitment, waiting lists) which are causing disillusionment amongst staff and staff are beginning to re-evaluate their career choices and willingness to engage with QI.

*“An awful lot of people re-evaluated their job in their heads, therefore, they realised that their enthusiasm and willingness to go the extra mile has been blunted and therefore, they are much less motivated to engage in future quality improvements”.*

Similarly, Daniel highlighted the toll Covid-19 has taken on healthcare staff and how they are less inclined to engage in change-based initiatives.

*“Staff have went above and beyond, and continue to, but week on week, as a manager, you are seeing their stress, you are seeing increased sick leave. And that's where when we are implementing change projects, I think you have to be very mindful of that, you know, change fatigue”.*

Michelle emphasised the concern around facilitating additional training for healthcare workers due to their high levels of exhaustion and lack of resources, predominantly time. Michelle noted the staff who are genuinely interested in QI and engaging in continued learning, are doing so in their own time outside of work.

*“The unfortunate thing we're hearing which from a staff wellbeing point of view is a bit concerning, is that a lot of staff, particularly those who are interested in quality improvement are not doing any training. If they are doing anything, it’s in their own time at home. That's... not what you want to hear, you know”.*

David expressed the need for a fundamental shift in how QI is presented in health services: it needs support from management, co-designing initiatives with front line workers, key personnel must be involved, people’s voices must be heard, a sense of ownership must be felt, accountability should be held, outcomes must be measured, data has to be collected, and buy-in must be sought.

*“I think there still needs to be a wider, sort of fundamental shift in terms of how staff are empowered across the health system to be able to make changes. And going back to you know, how a Quality Improvement is it going to work, it's only going to work if people feel that they can own it and if they're able to make those changes at the front line level”.*

Further, David spoke to the issue that it isn’t enough to have QI championed within one department. QI buy-in must be sought, encouraged and promoted throughout the entire organisation.

*“There's a lot of improvement work going on, and I think the challenge that we have is that it's quite siloed. So you have sort of individual departments that are doing, you know, doing a really nice project but it's just within one team, it's just maybe within one department, and what we're missing at the moment is process for pulling those together and sort of learning from all the different projects”.*

As expressed by David and Michelle, due to the lack of capacity and support, some healthcare workers feel they are unable or unwilling to engage with QI as it is seen as an add-on to their role. Jake similarly highlighted the difficultly of acquiring support for QI initiatives.

*“I’ve looked for support, it's not really forthcoming, you'd have to be banging on doors, and you know, harassing people to get that kind of support and I just don't have the time. I’m so busy chasing my tail I don't have time to do risk assessments, quality improvements, it's really hard you know, and so I find that very frustrating”.*

Tangible support and resources were deemed to be required to support staff, not just *“lip service”* as Sabrina and Gavin explained. Michelle shared the effect of a lack of support and resources on engagement in QI efforts.

*“We're finding it extremely difficult to get staff engaged in our QI programs, the education programs, because they just don't have the capacity to do it. Covid is still fairly real for a lot of frontline workers, you know. So that's an impact we absolutely are seeing. They don't necessarily have the capacity to engage in QI projects. It's like almost QI is on hold. That's what we're seeing at the minute”.*

## Discussion

The objective of this study was to explore the sustainability and resilience of not only the changes implemented by healthcare staff during Covid-19 in Ireland, but the resilience of the healthcare staff themselves through the various waves of Covid-19. Through using a narrative approach, four key themes were evident from the data: (i) From fear to exhaustion; (ii) maintaining person-centred approaches to care; (iii) Covid as a medium for change and; (iv) staff pride, resilience and appetite for QI. Overall, the key findings indicate that consideration of health care worker’s capacity to engage with QI must be considered when planning new initiatives. This also includes considering how hierarchies can act as a barrier to change, and the frustration this can cause amongst healthcare staff. Further, the experience and expertise of frontline health care workers must be engaged during the ideas and planning stage of any initiative. This partnership in planning will aid in creating an organisation that embraces the values of QI, and is change-ready, as it is not enough for a team to embrace QI, it must be embodied by the whole organisation.

From analysing the data within the themes, three main discussion areas have been identified: (i) integrating learning into policies and practice; (ii) the role of collective leadership and devolving/sharing power; and (iii) key drivers/factors that promote sustainability of QI interventions.

### Integrating learning into policies and practice

Ireland’s hospital system had some success in managing the additional burden of Covid-19 [33]. Although the pandemic exposed and exacerbated many of the long-standing issues within the Irish healthcare system such as capacity deficits, long waiting lists, overcrowding and poor infrastructure, due to the rapid innovation and change that took place during the pandemic, initiatives were fast-tracked that directly or indirectly combatted issues such as access to services [33]. This has resulted in initiatives, systems and processes that are now in place which have the potential to begin to effectively manage issues that the health system was experiencing pre-Covid. Without the emergency nature of Covid-19, some participants expressed that the projects or initiatives would still be in the development stages, waiting to pass through a multi-layered decision-making hierarchy.

However, the psychological state of individuals plays a role in their ability to buy-in to an initiative [34]. In order for people to find motivation to engage with initiatives in work, they must know that there is a return on personal investment and involvement in initiatives, possess an awareness that it is safe to fully immerse within the initiative without negative consequences and, have knowledge of the necessary physical and emotional resources that are available to support in the role [35]. As expressed by some participants, there is a worry that frontline healthcare workers may feel disenchanted and disengaged for the foreseeable future when it comes to QI, as they feel that they have nothing left to give due to the high levels of exhaustion [1–3]. Further, the notion of QI as being an extra add on to their work, as opposed to a fundamental way of working, exacerbates the challenge of obtaining buy-in to QI projects from frontline healthcare workers [36].

An engaged workforce consists of staff who feel valued, listened to, and are provided with the tools, resources, and skills to carry out meaningful work. It is vital that organisations access the unique knowledge of frontline staff and that their voices are heard across the organisation and used to inform improvements, ensuring that the learned experiences are integrated into polices and practice and QI becomes a fundamental way of working [37]. Such learned experiences can be integrated into policy by considering allocation of funding to specific QI training and resources and protecting staff time to promote engagement in quality improvement. Enabling this through fostering team-based approaches to working, building psychological safety in teams, and supporting collaboration will help serve to support cultures of learning and continuous quality improvement.

### Collective leadership and devolving/sharing power

Collective leadership has been described as “not located in one individual, but instead is a property of the team and something that can be shared to fit with task demands” ([37], p.3).The role of collective leadership to support, empower and entrust staff to identify and implement necessary changes is paramount to the sustainability of QI [9, 37]. In this study, participants enjoyed the more collective approach to leadership experienced during the pandemic and identified traditional hierarchies as a barrier to quality improvement and change.

In addition to feelings of burnout, stress and anxiety, moral injury has also appeared as a challenge for healthcare workers.6 Within the context of Covid-19, moral injury generally refers to the distress that can arise due to the necessary actions or lack of action from healthcare workers that violate their ethical or moral code.6 This occurs when healthcare workers may not have the resources or staffing to provide the best care possible and must make difficult decisions regarding fair allocation of scarce resources to patients (such as ventilators)0.6 However, moral injury initially referred to the issue of hierarchical power and betrayal of trust [38–40]. The emerging literature on staff experiences during Covid-19 utilising the newer definition of moral injury should not be overlooked. However, ignoring the original meaning of moral injury as a betrayal of trust within hierarchical power structures, ignores the influences of social and political context [40]. The newer individualistic approach ignores the systemic factors that could exist between burnout and moral injury. Specifically, a focus on betrayal-based moral injury allows for an insight into the influence of management and leadership, within healthcare settings and beyond [35]. Duplicitous speak can be considered an example of this. As shown in the results, with healthcare staff realising what is being verbally promoted on the surface by healthcare leaders, may not be what is actually occurring in practice. This can result in a myriad of complex feelings and strained relationships between healthcare staff and those in positions of leaderships.

This study further highlighted that as the emergency nature of the crisis wanes, hierarchical leadership styles are being re-established, despite the desire of staff who wish to retain the flattened hierarchy model that was established during Covid-19. However, it is evident that those in leadership positions must commit to providing top-down clarity and encourage bottom-up action [9]. Leaders should continue to create and champion an environment of empowerment, adequate resources, a commitment to increasing improvement capabilities, and a culture of continuous improvement for the long term [10]. Promoting such properties within a system can enhance its ability to function and encourage a more sustainable practice.8 Proactive, supportive and empathetic leadership was highlighted as being important to the success of QI projects within this study. However, even in areas where leadership support was absent or more of a laissez faire style, QI change efforts were still successfully implemented. Although, it must be highlighted that laissez faire leadership, a lack of support where support was not seen or felt, and the worry of returning to pre-existing issues affected the motivation of staff to further engage with QI.

Higher resilience, lower burnout, and higher wellbeing have been associated with personal and work related factors, such as meaning in life and the “collegial nature of the workforce”, respectively ([7], p.574). However, resilience among healthcare workers is influenced by a range of factors at the individual, organisational and societal levels [41]. This highlights the need for individual and organisational strategies for stress management, enhancing resilience, self-care [37], informal peer support initiatives and structured therapeutic interventions [23], access to professional mental health services,2 and supportive and proactive policy. Further, the importance of effective work design and operational pliability at the system level is vital to ensure resilience amongst the organisation and the staff within it [42]. However, due to the current individualistic idea of moral injury, individual support interventions have been favoured to alleviate feelings of guilt and shame. Such individualistic based supports were mentioned throughout this study, with participants reporting that helplines were put in place for staff. However, the participants could not comment on whether these helplines were beneficial as they were not utilised by the sample group. Feelings of anger and frustration must also be recognised when healthcare workers express such emotion towards their leadership (from local management to governmental structures) [35].

Thus, pro-active, visible, and effective leadership by board members, senior leaders, managers, and clinical leaders is essential to foster a culture of continual learning and improvement that demonstrates the values of the service, regularly listens to patients and staff, and seeks evidence of the quality of services [11].

### Key drivers/factors that promote sustainability of QI change efforts

Principles of sustainability must be embedded into healthcare practices through the fundamental teachings, training and education of healthcare professionals in order to ensure the continuation of the provision of high quality care into the future, despite any financial, social and environmental constraints [19]. Two key drivers/factors that promote the sustainability of QI interventions have been identified within the data from this study and the wider literature. These can be broadly categorised into i) individual factors; and ii) organisational factors [43].

#### Individual factors

Specific departments or units within a healthcare setting are complex and agile systems that evolve through an iterative process [39]. In terms of how sustainability of QI initiatives can be supported through more individual based factors, such as individual departments, three factors have been identified, including: i) understanding capacity; ii) continued learning and; iii) empowerment of QI champions.

##### *Understanding capacity*

Healthcare workers continued improvement efforts throughout the Covid-19 pandemic either unintentionally [11], or as a matter of need as seen in the increase of TeleHealth. However, QI can still often be seen as an extra add on to healthcare workers existing work responsibilities [44]. This has resulted in healthcare workers’ feeling as though they have not been allowed the time to process working through the Covid-19 pandemic, and are being expected to continue to work as though nothing has happened. This has created a sense that their exhaustion and frustrations have not been acknowledged [1]. Therefore, it is vital to understand the experience of healthcare workers who have engaged with QI throughout the Covid-19 pandemic and meet them where they are to understand their current capacity, to support and nurture their resilience and to learn from their experiences of what has worked well, despite the challenges encountered during this unprecedented and trying time.

##### *Continued learning*

To demonstrate that QI should be viewed as a fundamental part of the work healthcare workers engage in, continued learning amongst healthcare staff should be encouraged [45]. However, once again, QI facilitators that were interviewed for this study concerned about hosting additional training sessions for healthcare workers due to their feelings of burnout as a result of the Covid-19 pandemic.

##### *Empowering QI champions*

When staff examine the issues that affect them in their working lives, and know that QI initiatives may improve not only their experience, but the experience of those receiving care, encouraging staff to look at issues with a new perspective and brainstorming innovative ways of delivering care can be beneficial. By understanding healthcare workers capacity and a culture of continued learning, staff can begin to feel a sense of ownership of different QI initiatives that they are involved in [45]. However, when there is buy-in amongst one department of the benefits of QI and that department champions QI, buy-in may not necessarily be more easily sought from other departments within the organisation [46].

#### Organisational factors

Although individual factors can influence the success of a QI initiative, literature and indeed this study has indicated it is primarily organisational factors that influence the sustainability of QI [39].

##### *Organisational context*

Within the context of this study, organisational context can be understood as the “underlying systems, culture and circumstances of the environment in which an intervention is implemented” ([47] p.2). When an organisation values and consciously undertakes and promotes QI methods, a QI culture emerges. When an organisation values and consciously undertakes and promotes QI methods, a QI culture emerges. A QI culture consists of a work environment that is dedicated to moving away from imposing top-down solutions. Instead, a QI culture listens to the experience of those on the frontlines and service users. A QI culture ensures staff feel encouraged and empowered to implement small tests of change to improve their work, and where resources are provided so staff can engage in QI projects [48]. Ensuring that a QI culture is embedded within an organisational context is key to ensure that best-practices and a change ready environment becomes part and parcel with every-day work. Further, such a culture allows for a degree of psychological safety amongst staff so they feel a level of comfort, support and confidence to recognise that when problems arise, they can voice their opinions on how issues can be resolved. Moreover, a QI culture ensures that staff know that there is access to the expertise and tools that they may need to engage with QI initiatives. Further, it creates awareness around the support structures that exist to empower people. However, it is not enough for a culture of QI to exist within just one department at an individual team level, it must permeate throughout the entire organisation.

##### *Competing priorities*

When there is competing priorities within an organisation and within wider society, such as the Covid-19 pandemic, the focus of initiatives such as QI can be set aside until the other priorities are more manageable [11]. This therefore further underscores how essential a QI culture is within healthcare organisations to ensure that QI practices do not come to a sudden halt due to competing priorities.

##### *Resources*

In order for QI initiatives to be sustained, they must have the necessary supporting resources, including access to the relevant expertise to support with the initiatives, or where they are unavailable, avenues for staff to learn the techniques themselves to engage with QI. However, the limited availability of necessary resources within the Irish healthcare system have been exposed as a result of the Covid-19 pandemic. When a health system is facing long-standing issues such as capacity deficits, long waiting lists, overcrowding and poor infrastructure in the physical environment [31], assigning resources to QI improvements may not be a key focus for some people. However, as seen within this study, QI initiatives can often address and begin to overcome long standing pre-existing issues within health systems, and therefore should be supported, prioritised and encouraged.

The key drivers that influence the sustainability of QI initiatives can be divided into individual and organisational factors. Overall, it is essential to understand the capacity of healthcare teams and to harness their current appetite for change through QI, and for senior management to support and encourage healthcare teams to engage with QI by allocating resources to QI initiatives, and supporting a QI culture to become embedded within the organisational structure to combat issues that arise.

##### *Limitations*

It would have been preferable to acquire a larger number of participants to deepen the insights into the experiences of healthcare workers engaging with QI during the Covid-19 pandemic. However due to the busy schedules of healthcare workers, it was a challenge recruiting a wider sample at the time this study was conducted. Further, the sample consists of people from a relatively homogenous background, a White-Irish background. It would be worthwhile for future studies to ensure a sample of participants from a diverse background to understand the experiences of healthcare workers from minority backgrounds as their experience may have differed. As with all methodology, there are limitations to a narrative inquiry. A narrative inquiry possesses a fundamental weakness as it is retrospective, although, the reflective nature of a narrative inquiry could be argued to be an advantage of the methodology [49].

## Conclusions

A growing body of literature is exploring healthcare staff experiences of the work environment during the Covid-19 pandemic. As the waves of Covid-19 have declined, it is vital to examine how the feelings of burnout and disillusionment will affect engagement with and the sustainability of quality improvement initiatives. This study has helped to address this gap. Improvement work must be embedded within the culture of the work environment so that QI is relevant and accessible to those on the front line. Improvement is more likely to be successful and sustained if it is conceived and delivered in partnership and supported by bureaucratic hierarchies. This paper offers significant and novel contribution to healthcare staff’s experience of engaging with QI initiatives during Covid-19, and how the lessons learned can improve not only the sustainability and resilience of QI initiatives, but of healthcare staff themselves.

## Supplementary Information

**Additional file 1.** Interview schedule.

**Additional file 2.** Narrative summaries.

## Data Availability

The data that support the findings of this study are not openly available due to reasons of sensitivity and confidentiality, but are available from the corresponding author upon reasonable request.

## Abbreviations

QI: Quality Improvement
QPS: Quality and Patient Safety
GP: General Practitioners
PDSA: Plan-Do-Study-Act
HSE: Health Service Executive
