# Supplementary material for: A narrative inquiry into healthcare staff resilience and the sustainability of Quality Improvement implementation efforts during Covid-19
Source: BMC Health Serv Res. 2023 Feb 24;23:195. doi: 10.1186/s12913-023-09190-4 (PMC9949907; doi:10.1186/s12913-023-09190-4)
Supplement: Supplementary file 1 — Additional file 1. Interview schedule. [file 12913_2023_9190_MOESM1_ESM.docx]

**Interview Schedule**

*AIM:* Explore staff resilience and the sustainability of QI implementations/efforts.

*Introduce self, explain research aims briefly, check interviewee has read the information sheet, answer any questions, ask interviewee to sign consent form and advise they can keep a copy of information and/or consent sheets. Request permission to record - explain processes.*

Background questions:

1. Can you tell me a bit about your professional background / time as a X professional?
2. What is your current job? How long have you been in this role?
3. Do you work in a team/teams? What are your responsibilities in this team/s?
4. Have you led and/or been involved in implementing any service improvements in the past?

Substantive questions:

1. Did you change your (or) come up with new ways of working during the COVID-19 response?
   1. What was it like before?
   2. What is it like during COVID-19?
2. Who was involved in the change?
   1. Who had the idea? Who initiated it/collaborated on it?
   2. Who supported it?
   3. Any support from formal leadership structure?

*Why was this the case do you think?*

- 1. Any involvement from patients or non HSCP?

1. What was the aim of the change?
2. What did you do?
   1. What motivated/inspired you to make this suggestion or initiate the change?
   2. How confident were you that you could execute the change? Was this level of confidence different due to COVID-19?
   3. What was the process to obtain approvals for this? Was it different pre-Covid?

*What makes you say that? Can you give me an example?*

- 1. How did you gain the support of colleagues to implement the change?
  2. Did you use any QI tool/framework? Did you employ an iterative process, adapting and learning as you went along?

1. Was there anything different about how you worked with colleagues to implement the change/initiative as compared to how you usually work together? What were the key differences? Did people adopt different roles/behave differently to how they normally do?
2. What were the outcomes (staff/patients)?

*What impact have these changes had (on staff, patients, team performance)?*

- 1. Impact on team dynamics?
  2. Impact on productivity/efficiency?

1. Were/Are the outcomes being monitored?
   1. If yes, HOW?
2. Was the change/initiative successful in your opinion? If so, what were the main factors that contributed to this success?
3. Has this process/initiative continued post-COVID response? Has it changed in anyway? If so, how?

*Why do you say this?*

1. During the pandemic response, staff have been under pressure to maintain access to services and continue to deliver high quality and safe care. In your experience of working with colleagues, how are staff feeling at the moment? Has this changed over the course of the pandemic? What makes you say this?
2. Have there been any additional supports for staff during this time? (if yes, seek examples) what helped staff cope during this period?
3. What impact do feelings like this among healthcare staff have on efforts to make changes and improvements to care delivery? Can you give me an example?/What makes you say this?
4. What do staff need at the moment in your opinion to effectively manage and sustain quality and improvement initiatives implemented during the pandemic response?

Closing:

Was there anything else you wanted to mention today that I haven’t specifically asked about?

Prompt for any other reflections on involvement/implementation of the initiative that haven’t been specifically asked about.

***Thank participant for their time and for sharing their experiences.***
