# Supplementary material for: A narrative inquiry into healthcare staff resilience and the sustainability of Quality Improvement implementation efforts during Covid-19
Source: BMC Health Serv Res. 2023 Feb 24;23:195. doi: 10.1186/s12913-023-09190-4 (PMC9949907; doi:10.1186/s12913-023-09190-4)
Supplement: Supplementary file 2 — Additional file 2. Narrative summaries. [file 12913_2023_9190_MOESM2_ESM.docx]

| **Clara (nurse, lecturer)** |
| --- |
| I was working in education and when Covid hit I was redeployed to the frontline. I was happy to do that because at the end of the day I am a nurse and patient care had to be delivered. Initially there was a lot of stress and uncertainty around the new infection prevention protocols and people fearing that they may catch Covid. The structure of our day changed; what we wore, how we engaged with each other, how we interacted with patients, how we ate, how we came to work, how we went home.  During the waves of Covid there were days when there was no staff available. Some staff were absolutely exhausted but couldn’t take a break or leave because the lack of staff. They also didn’t want to leave or abandon their patients. It was nurses who remained at the bedside when patients were dying, and nurses who questioned if they had done enough for their patient. We felt that we were not supported as nurses and felt let down by our superiors and other disciplines. However, we did find solace in experiencing the same difficulties and emotions. We are proud that we stood up and delivered care when it mattered most and realised just how flexible and adaptable we can be.  Although Covid was a challenge, it shone a light on what can be achieved in a short space of time by having key personnel collaborate and share decision making spaces. I think it’s important that we draw on the experiences during Covid as a benchmark to what can be done when we do take away a lot of the layered decision making and red tape. Covid has highlighted what initiatives need to be at the forefront of any sort of quality drive in an organization and I think it must be down to having our patients and residents interests there and how we can ensure that they are safe and that our staff working with them are safe as well. |
| **Daniel (physiotherapist clinician)** |
| I began my role as a physiotherapist clinician just five months before Covid hit. During the initial weeks of Covid, there was a high level of apprehension and fear amongst staff. Some of my staff expressed their concerns around having enough PPE, having the right skills, if they were high risk, if they were pregnant, and if they were going to bring the virus home to their elderly parents. I knew there was going to be a lot of changes in how we operated, for staff and for our patients, particularly our respiratory patients.  As a result, we had to adapt our TeleHealth service, so our outpatients service changed. Higher ups only wanted the high risk patients coming in house, so we had to quickly adapt to a TeleHealth service for OPD. It all seemed to happen overnight, but there was a lot of depth and planning to it albeit it fast. It wasn't easy but we were able to keep our services going throughout and that was important for us, to limit disruption for our patients. Having a culture of Quality Improvement within our organisation helped with the quick change of our outpatient service. As a manager I am a strong advocate of QI and believe that line management, senior management and all clinicians should be encouraged to engage with QI as an everyday practice. Senior management supported the initiative and I think a lot of people were more open to solutions and collaborative brainstorming. Everybody was listened to and hopefully we won’t go back to the pre-Covid way of ‘this is how we do stuff’, with no room for collaboration.  Although staff felt that they're being listened to and they're being empowered to change things themselves, I think they are now totally exhausted. We were told this is going to be a marathon, but it's been an ultra marathon. As a manager you see their stress, you are seeing increased sick leave. I think you have to be very mindful of change fatigue when implementing change projects, and be mindful that everybody approaches change differently. We have to remain safe within the resources that we have because we still have continual sick leave in terms of covid and isolation, so it's constantly changing daily, hourly even. I just hope that the policymakers will realise the importance of QI because such good change has been done with Covid. It has given people a voice to empower them to actually test things. So I just hope we don't go back to that hierarchy where all ideas are kind of shut down, and I hope that they continue to support QI going forward, because it is the only way that people will improve standards. |
| **David (consultant microbiologist)** |
| My main role is as a consultant microbiologist. Although I am also a clinical lead for quality improvement, that aspect of my job has taken a back seat due to Covid. However, in the last five months, I think we're starting to move back towards something resembling normality and I am beginning to prioritize the quality improvement work a bit more.  In spite of Covid, improvement work was on-going. However, the challenge of siloed work remains, with individual departments doing fantastic work, but not communicating with other departments who may be doing something similar, or who may benefit from each other’s work. So we're trying to develop wider QI faculty to include a team to support that sort of approach in terms of supporting QI and shared learning. However, Covid allowed for the realisation that we can implement change quickly and I think that's a really important positive out of this.  Unfortunately individual and system resilience has been sorely tested. There has been a sense of being in the trenches and being under huge pressure for the last two years. I think it's very difficult for people then to engage with quality. These are seen as optional extras for which people say ‘well I just don't have time for that, right now’. Staff are exhausted and burnt out. I think there's also a lot of people that have a concern that when we talk about returning to normality, it’s the realization that normality may not be the most efficient, high quality health service. We still have the same problems in terms of resourcing, hierarchies and outdated management approaches that were there before the pandemic and to a certain extent, got pushed to one side. You have that sense of hope but it's balanced out with this sense of ‘oh god, all that stuff that we had to deal with before, it's still there’. I think there still needs to be a wider, sort of fundamental shift in terms of how staff are empowered across the health system to be able to make changes. And going back to how quality improvement is going to work, it's only going to work if people feel that they can own it and if they're able to make those changes at the front line level. |
| **Gavin (clinical audit facilitator)** |
| I’m a clinical audit facilitator in a hospital group and experienced a variety of changes at the beginning of Covid. When Covid hit, I was no longer working in the office or having face to face meetings. It felt like there was a hiatus before the move to online platforms to facilitate hybrid meetings. Unfortunately for people joining online to those meetings, they felt excluded from what was happening, they didn’t feel heard and they were not included in side bar conversations. In our organization that message really did not percolate to senior management, despite being reminded of it, forcefully at times. And everybody went “oh yeah yeah, we must do something about that”, and nothing was done.  Our organisation struggled with quality improvement during Covid and used no real framework. Like a lot of quality improvement things, we got 75% of the way and we lacked the ability or the motivation to go to the final hundred percent. As an example of that, outpatient became virtual clinics and we were inundated with teams wanting to do audits on the satisfaction with virtual clinics. We kept asking people whether or not they prefer virtual clinics or physical clinics, but we weren't offering them physical clinics. So we're offering them a choice of two on the menu, on which one was off the menu. We got answers back which people said they'd love a mixture of them. That didn't work. Changes during Covid happened quickly but there was no structured process to the changes. I think people just made it up as they went along. I don't think that there was a conscious approval system for any of this. However, Covid did highlight certain defects in the organisation, if you see that as a good thing in the sense that it shone a light into dark spaces and one of those dark spaces has been the concept that we kept telling ourselves “we're patient centered”. The hospitals all over the country have “we're patient centered” as a kind of value that they cherish dearly. It actually got senior people to admit, behind closed doors, at times, “really we’re an awful lot better without visitors, aren’t we?”. And you kind of go “you can't say that” but that to me clarified a duplicitous speak in organisations. And so I think frontline staff began to realise that what is said might not be the value in action.  Staff are exhausted. People need holidays now, and they are beginning to get them and they're beginning to come back to their old self but after two years, that took its toll. An awful lot of people revaluated their job in their heads, therefore, they realised that their enthusiasm and willingness to go the extra mile has been blunted and therefore, they are much less motivated to engage in future quality improvements. I think the government's thousand euros thing has dragged on and annoyed people. I think even now, when it's coming to being paid, understandably, it had to have limitations, but people are thinking “because I wasn't dealing with frontline people and I'm not going to get it, I'm not going to bother”. Again, another thing, the most dangerous thing in an organisation is the subtle withdrawal of enthusiasm, and I think that's what we're going to see for the next few years. You need human interaction. They need people to come along and tell them that they're doing a good job, and not just kind of lip service but actual tangible resources and making their job easier and they need acknowledgement and they need the space to do these things, so they need the hierarchy to give them permission to do these things. And I think again, there are elements, because people have seen a new way of doing things and they've seen how things can change overnight, so therefore there is that fertile seabed there for them. But I think leaders will have to do an awful lot more to earn the trust and respect of frontline staff. |
| **Jake (paediatric dentist)** |
| I am a paediatric dentist and at the moment, there should be two people doing what I’m doing and I’m currently the only one, so one of the positions is vacant. So I’m kind of just keeping my head above water at the moment, so it's been quite tricky and that's been basically since covid started to be honest, because my colleague retired, about a year into covid, but for the most of the first year he was working off site which in dentistry isn't very productive. When Covid hit, we tried to keep people as separate as possible and we found that very hard, because we were used to being a very close team, a very small team, seeing each other every day, working very closely together, so we did find that hard.  A QI initiative that we are engaging with that is not specifically related to Covid but was forced out of Covid to attempt to manage the waiting lists is the TORC initiative (Triage Of Referral Clinic). It’s a way of filtering the referrals and making some actions on them so that more information is collected before the appointment. My team and I struggled to receive support from senior management. They didn’t stop us doing it but there wasn’t much in the way of active support or encouragement. You can only seem to get resources for these things after the events, so you've got to kind of do it without funding, without extra help and then, when you show it works, then they give you some extra help. We didn’t consult a QI framework for this initiative even though I’m aware of the change framework with the HSE. I haven't had support on that and I’ve looked for support, but it's not really forthcoming. You'd have to be banging on doors and harassing people to get that kind of support and I just don't have the time. I’m so busy chasing my tail I don't have time to do risk assessments, quality improvements, it's really hard and so I find that very frustrating.  Most of my team are glad to be back at work. I think I am very lucky in the sense that my team are keen to implement change. We just find it hard to actually consistently keep up with the change, you know with whatever idea it is because you fall back into your old ways, your habits, because I suppose we get this decision fatigue. I don't have a person I can say ‘well this is your responsibility. I want you to take your foot off the pedal on the other bit that you would be normally doing and just focus on this’. I don't have spare people to do that. Rationing my energy is one thing that I’ve learned and I’m not sure if it's quality improvement or actually quality disimprovement, but I suppose what it means is that it's keeping a sustainable level of care so being good enough almost all of the time is better than being really brilliant sometimes and then down to zero, which then isn't really helping anyone. |
| **Maggie (quality facilitator)** |
| I work as a quality facilitator however my role changed during Covid. I became involved in building a tracking system so that management would know where every nurse was at every time, so if someone rang in sick they could see exactly who they were electronically and see who and where they were immediately. I suppose my experience is a little different. It was a very exciting time from my teams perspective. We weren’t frontline so we didn’t have the problems they were running into. It was very motivating and innovative and a whole new team came together. We hadn’t worked together in that capacity before so it was a brand new time in a completely new department that was set up, so I really enjoyed it, it was a very exciting time.  The nurse tracking system was in the works before Covid hit but it was fast tracked due to the pandemic. The system was a success and is still used now. We measured every single day who was using the system and who wasn’t. We put a plan in place for each individual area. Using early adaptor theory and QI methodologies such as agile project management, lean methodologies, CQI (continuous quality improvement), PDSA (plan do study act), we identified areas that we felt would come in easily and we had enthusiastic staff members to help us get through those early stages. I think everyone was very focused on supporting the frontline, everyone was united and wanted to get this right. Everyone pulled together as hard as they could because of the situation we were in. It had to happen, we knew we were in a crisis, we knew it had to be brought over the line. The system was successful due to the QI culture which had to be built into the organisational structure, it was on every single agenda, every single minutes, every single terms of reference. You have to build it the whole way through. It has to be a core part of everything as opposed to a separate entity.  Although my experience was unique and I knew how privileged I was to be working within that team and it was something that I’d be able to tell my grandchildren about, I know that it is because I wasn’t on the frontline. I was safer. Those at the frontline now are exhausted and feel like they don’t have the capacity to engage with QI. |
| **Michelle (quality and patient safety educationalist)** |
| I am a quality and patient safety educationalist and I was redeployed to the contact management programme during the pandemic. So it was a complete start up. We were applying rapid pdsa cycles to everything we did. We started off trying to develop a very basic training program for contact tracers and developing scripts that they would use as they were making the contact tracing calls. The evidence was changing all the time, so we had to keep changing the resources, be it the training materials or the scripts and testing them. And though we might not have done it consciously, unconsciously we were trailing things, testing them, making improvements, and that was just something we did, probably because it is the way we do business. Our manager came with us and our director so it was an entire directorate that moved, so we certainly had support. The managers themselves were rolling up their sleeves and getting on with the work at the same time. We had good project management as well and from a leadership point of view that was proved to be excellent. I think we were lucky in a way in that team. Everybody understood kind of the language of “let's test this” or “are we ready to pilot this?”. People were comfortable and knew each other, so there wasn't necessarily that reluctance to get stuck in as you might have with people who didn't know each other.  We had a very supportive, open and collaborative culture within the team. However, even without any crisis there'll be people who excel in that and there'll be people who find that really challenging. Overall people really got stuck in and supported each other. I also think we bypassed a lot of bureaucracy in that time. We escalated a lot of things and doors were open to us to enable us to do the job. I think we question things more now and say “well look, why does it take six months to get something approved when it could be done shorter?” So it has given us an insight into what can be done when your back is to the wall. I hope it doesn’t revert back to the way it was before Covid.  I think staff are tired. We're finding it extremely difficult to get staff engaged in our QI programs, the education programs, because they just don't have the capacity to do it. Covid is still fairly real for a lot of frontline workers. It’s like QI is on hold. That's what we're seeing at the minute. We would monitor engagement in our QI programs and pull reports and we can see that there certainly is a dip in take up at the minute, which again, it's kind of worrying. We are working with quality and patient safety leads out in the system trying to see how can we encourage people to do our programs. And the feedback we're getting is that people don't have the time to be coming away for a day, they want learning in small bite sized chunks and flexible modes of delivery. I think the biggest issue is a scarcity of resources. Staff can’t be released to engage in any type of learning and development. So unfortunately we're at the bottom of the list from a quality improvement point of view when you're trying to encourage somebody to do anything. I’m not sure what the answer is yet and that's what we're trying to explore at the minute, what can we do to support staff to try and get them engaged again? What can they their managers do? But it’s going to be a challenge, I think, for the next year, at least. |
| **Sabrina (service improvement lead)** |
| Before covid, I was a service improvement lead in a hospital group but I was redeployed in the early months of the pandemic to go to a hospital to help manage the immediate crisis. Once the dust settled later in the Autumn of 2020, we had to look at how can we get back to doing our improvement work and get back on track with that. We found it challenging to move our improvement work online due to the resistance that can come with change. I was once again redeployed to the national Covid test and trace a year ago and that is where I am currently working.  During the Autumn of 2020 when we were working on QI, I found that it was tricky to organise online training. It was more time consuming, we needed more prep, we needed more facilitators because it was trickier to manage. Fortunately there were positives as it worked really well for this particular group because they were really diverse. They wouldn't have all come into a room for a week because people aren’t going to travel in from all parts of the county to sit in the hospital room for a week or in a meeting room, so that actually worked to our favour for that particular piece of improvement. Because people dropped in and out, more people engaged. I think the eventual success of continuing QI work was down to our QI culture. We would often refer to the lean framework. Although Covid was a challenge, our communication as a team is better because we had daily huddles and check ins. We involve people with QI across the team, from the most junior member to the most senior member. But that wouldn't be widespread necessarily. There are a lot of teams that I work closely with who feel the exact opposite. They really just feel that they don't have a voice, they don't have an opportunity, they don’t feel listened to. I think that's a big challenge. In the HSE we have our value statement and that's really good but that doesn't necessarily translate into individual people's experience in the place they work.  At the beginning of the pandemic, staff were very stressed, they were working long hours, working weekends, working extra time. We all felt a bit burnt out. Whereas now, it depends where you work. For some of us it has levelled off and we've got through the worst of it and we're in a stable state. For some frontline services, they’ve never really got that break, they haven't really had that recovery time because the intensity is still there in a different way. Now they’re back to the old issues of trolleys and waiting lists. So there’s a lot of burnout and there's a lot of disillusionment among staff, but not everywhere. In regards to staff engaging with QI, people's motivation to go above and beyond for another step beyond the beyond that they've already gone, many would see that as a step too far, and probably not feel terribly motivated. That being said, the other side is now that people have got used to working differently and have seen how change can happen quickly and how you can make things better, then that could open up a willingness. We've done a lot to try and talk about person centered care and that staff are valued, but it has to be real and meaningful and not just words, not just lip service to staff that they are being valued. It's approaching change in a way that staff actually experienced that feeling of being valued as opposed to just being told that they're valued. |
| **Sarah (community health care network manager)** |
| I was voluntarily redeployed at the beginning of the pandemic because the role I was in was a Slaintecare project, so it was suspended. I moved in to establish the Covid 19 testing services across the CHO. I spent about nine months working in the early stage from March 2020 just setting up and establishing our covid services across the CHO and then I returned to my role and picked up the project that I was working on and took the experiences from Covid into the role.  One QI project we are working on is an electronic referral pathway between our GP colleagues into primary care. This project was really enabled by covid. We started this project in late 2019, pre-covid. And at the time we didn't have a connection between the HealthLink, between GPs and primary care services. We did come up against a little bit of a brick wall, where we were told from our communications department that we couldn't actually connect the two. But at the early days of covid, the referral pathway was established and every GP in the country had access through their HealthLink service. It was really beneficial as it pre-populates a lot of the client data onto the referral form, so it really reduces their workload. It reduced missing information, missing data. And the key enabler for us was the fact that now every GP had access, so all we needed to do was connect the primary care side of things. So we just set ourselves up as a referral receiver the same as you would have in a test Center or a covid assessment hub. It couldn't happen before covid and then when we went to pick up the project again in March 2021 it was possible. What I’ve learned in the last two years is that quality improvement is everything we do, every day. And it's just about putting a structure around it just to demonstrate what we're doing and why we're doing, so that we can look back and say what we've achieved.  I think there's a bit of a divide at the moment around how staff feel. If people felt that they weren't supported during the early stage, some people may withdraw a little bit and come to that point around you know, ‘why are we continuing? I'm coming in, I can do my work and that's as much as I can cope with at the moment’. And they see quality improvement as another job on top of what they're already struggling to manage, whereas I think it’s key to really shift into seeing that quality improvement is just a part of what we do every day. Some people are ready, they're on board, they're ready to run with it. Some other people need a little bit of convincing and other people need to see the outcome before they’ll come on board, and that's absolutely fine, we're just meeting people where they're at. It's about mental capacity in a physically busy environment. If you've got a huge waiting list or a huge case code, people don’t have the mental capacity. It’s about carving out that little bit of mental time for someone to think about where they want to go next and balancing that with the demands of our key performance indicators and our waiting lists and our external media pressures of the state. We need permission from senior management or leadership or local leadership to carve out a little bit of protected time to just think outside of your day to day busy work and that to me is the fundamental. |
| **Stacey (service improvement lead)** |
| I work as a service improvement lead in a hospital. One change initiative that I was involved in during the pandemic was shifting our entire outpatient service. We would see about 30,000 contacts annually in our outpatient department and we shifted that entire model onto a Telehealth platform within about three weeks of lockdown back in March 2020. We've used quality improvement methodology over the last two years to embed now a hybrid model and so that can flex up and flex down depending on if there's changes around access to the hospital. It also has allowed us to and expedite services that we thought about creating way back when, but there was always barriers and then suddenly with covid there was no barriers, because everyone was happy to try and get stuff done and so we've been able to hold on to a lot of what our patients would have wanted.  I think one of the things that made the switch to TeleHealth so successful for us is that we were change ready. And we try and always be a change ready environment and use the relevant change and QI frameworks and methodology, that's the culture we try and embed. So we try and look around corners and see down the road for what's potentially coming. So we had already been doing work around shifting some of our models of care onto an online platform or embracing more of Telehealth. So then when covid hit, we pulled everyone together and quickly determined “right what can we shift next week? And how do we do that?”. We felt relatively supported during this time from senior management. It was support in that they didn't get in our way but they probably didn't come looking to see if there was anything else we needed or anything else that needed to be shared. Related to that, covid did remove a lot of the red tape around passing changes. Pre-covid there were lots of formal layers; there's a project request form, there's a business case template, there's a protected pros and cons piece that you have to think about and then you would typically have to line up lots of conversations to then present at a formal meeting that you have to wait for your slot and that could be months down the line, they might want you to do ethics because it's service improvement, so there's lots of different tick the box exercises going on.  At the minute there's a lull in terms of covid. I think now people are beginning to realize just how tired they are, and yet we have pressures to kind of get back to improving health care. And everyone's a little bit like “oh God I'm not sure I have the headspace for this, I'm exhausted”. We need an honest acknowledgement of where staff are at and resources to not only make sure that staffing is at an acceptable level, but resources to support work to take time away to look at improvement. During covid, we got some extra posts approved, but they’re all gone now. We're not really back to normal staffing levels because we've still got some staff off now as covid is still present in the community. We still have some staff coming and going all the time and we're having to cope with those absences without any extra support. But I think there's a lot of reflection in terms of pride and sometimes the surprize around what was possible, what we did, what we're holding on to and so trying to bring forward all of the positive learning and use that to keep plugging away at just making things a little bit better incrementally as we go. |
| **Tom (cardiac physiologist, lecturer)** |
| Up until February 2022 I ran a cardiac physiology department in a hospital. During covid, I quickly realised we had to re-evaluate how we ran our day to day operation and this included breaking the team into pods and implementing a four day work week, which has been sustained. However, we no longer work in pods.  The four day work week has been sustained because it works for the service and it works for the team. We agreed on ground rules early in the process and tried to tease out all the challenges that might happen. It was sustained because the team themselves were involved in designing how it would work with a recognition of it needing to work for the service as well. I think getting that balance right is important but I think one of the things we are starting to lose that we had during Covid is the ability to rapidly develop things. Giving local line managers the autonomy to do what works, I think we're losing that a little bit, and also the rapidity that we could change things has started to go back. I also think we are almost afraid to make mistakes in healthcare and with service change. Everything almost has to be perfect. And so, if you're going to change something, you almost want it perfect before you change it. I think part of it is the hierarchy has returned a little bit. When we broke into pods the hierarchy was flattened. And people had to step up and take responsibility and they were allowed space to grow into that. I try to prevent myself going backwards in the way I manage people but invariably you do. And I think there was so much rapid change happening during the initial stage covid that you really had to let people just do what works.  I think staff are now exhausted, but I don't think they're necessarily exhausted from covid. I think they're exhausted because of our situation of being short staffed. We have run some unsuccessful recruitment campaigns, there’s just a shortage of cardiac physiologists. It’s not only nationally, but also internationally. So it's a challenging recruitment environment, and I think that's the thing more so than covid, what's causing the sort of exhaustion. I worry about if the recruitment situation doesn't improve, if we don't get staff on board, it could lead to some demoralisation but generally they seem to be very good as team, like we always had a great team and mind each other, and that seems to have persisted which is great to see. |
